# Validity and Reliability of the Urdu Version of the McLean Screening Instrument for Borderline Personality Disorder

**DOI:** 10.3389/fpsyg.2021.533526

**Published:** 2021-08-20

**Authors:** Khadeeja Munawar, Muhammad Aqeel, Tasnim Rehna, Kanwar Hamza Shuja, Faizah Safina Bakrin, Fahad Riaz Choudhry

**Affiliations:** ^1^Department of Psychology, Jeffrey Cheah School of Medicine and Health Sciences (JCSMHS), Monash University Malaysia, Subang Jaya, Malaysia; ^2^Department of Psychology, Foundation University, Islamabad, Pakistan; ^3^Department of Applied Psychology, National University of Modern Languages, Islamabad, Pakistan; ^4^Department of Psychology, Capital University of Science & Technology, Islamabad, Pakistan; ^5^School of Pharmacy, Kumpulan Perubatan Johor (KPJ) Healthcare University College, Nilai, Malaysia; ^6^Department of Psychology, Kulliyyah of Islamic Revealed Knowledge and Human Sciences, International Islamic University Malaysia, Selayang, Malaysia

**Keywords:** adults, Urdu, confirmatory factor analysis, Pakistani, psychometrics, individuals with cardiac problems

## Abstract

**Introduction:** Although the translation and the validation of the McLean Screening Instrument for Borderline Personality Disorder (MSI-BPD) are performed in various languages and samples, no study has established the validity and reliability of the Urdu version of MSI-BPD in individuals with cardiac problems.

**Materials and Methods:** The Urdu version of the MSI-BPD was prepared through the standard back-translation method. The translation and adaption were completed in four steps: forward translation, adaption and translation, back translation and committee approach, and cross-language validation. The sample, selected through the purposive sampling method, comprised of 150 adults with cardiac problems (men = 75 and women = 75), with an age range of 23–78 years (*M* = 55, *SD* = 10.6). The Cronbach alpha reliability and factorial validity of the MSI-BPD were assessed through confirmatory factor analysis (CFA) and Pearson correlation analyses. Internal consistency and test–retest reliability (at 2 weeks' interval) were used to evaluate the reliability. Statistical analyses were performed, using SPSS (version 22) and Structural Equation Modeling (SEM) software.

**Results:** Preliminary analysis revealed that the overall instrument had good internal consistency (Urdu MSI-BPD α = 0.79; English MSI-BPD α = 0.77) as well as test–retest correlation coefficients for 15 days (*r* = 0.94).

**Conclusions:** Findings suggested that the MSI-BPD, with important limitations, can be used as an effective preliminary screening tool to measure BPD in Urdu-speaking adults with cardiac problems. Further validations should be conducted to make the translated version of the MSI-BPD an appropriate tool to screen BPD in hospitals and mental health care settings.

## Introduction

About the association between physical health issues/conditions and borderline personality disorder (BPD), earlier studies showed a relationship between BPD and various deleterious physiological outcomes such as the enhanced risk of severe chronic illnesses, including heart disease, arthritis, and diabetes (Maiß et al., 2021; Sigrist et al., 2021). Nevertheless, few studies have explored the relationship between BPD, health care utilization, and medical comorbidities. Latest empirical evidence has demonstrated associations between the presence of BPD (or symptoms) and higher rates of cardiovascular diseases, liver diseases, hypertension, venereal, and gastrointestinal diseases (El-Gabalawy et al., 2010; Benjamin et al., 2019; Virani et al., 2020; Cavicchioli et al., 2021). However, studies exploring the direct association between the presence of symptoms of BPD and cardiovascular diseases in medical samples are still scarce, and present empirical inquiry was conducted to add to the body of literature in this regard (Sher, 2005; Powers and Oltmanns, 2013; Gale et al., 2014; Maiß et al., 2021; Sigrist et al., 2021).

The term “borderline” was first coined by Adolph Stern in the United States in 1938 (Stefana, 2015; Linehan, 2018). Stern (1938) suggested this term for a group of patients who “fit neither into the psychotic nor in the psychoneurotic group” as these patients “bordered” on other settings (Zandersen et al., 2019). Afterward, Kernberg proposed the term “borderline personality organization” (Kernberg, 1975) to refer to a consistent pattern of functioning and behavior categorized by uncertainty, reflecting bothered psychological self-organization (Berenson et al., 2018; Friedel, 2018). Borderline personality disorder is characterized by a persistent pattern of volatile interpersonal associations, intense emotional imbalance and psychological distress, persistent suicidal predispositions, extended identity disturbance and self-image, and impulsivity (Fusco and Freeman, 2004; Kreisman and Straus, 2004; American Psychiatric Association, 2013; Kreisman, 2014; Schweitzer, 2015; King, 2017; Sperry and Peluso, 2018). According to the Diagnostic and Statistical Manual of Mental Disorders, Fifth Edition (DSM-5), a diagnosis of BPD is based on: “(1) a pervasive pattern of instability of interpersonal relationships, self-image, and affects, and (2) marked impulsivity beginning by early adulthood and present in a variety of contexts…” (American Psychiatric Association, 2013, p. 663).

Borderline personality disorder is one of the most prevalent disorders in clinical settings, and evidence suggests that it is more common compared with schizophrenia (Gunderson, 1984; Zimmerman et al., 2005; Linehan, 2018; Zanarini, 2018; Chapman et al., 2019). The past empirical evidence showed 3–6% prevalence of BPD globally (Swartz et al., 1990; Torgersen et al., 2001; Regeer et al., 2004; Grant et al., 2008; Lenzenweger, 2008; Merikangas et al., 2011; Shen et al., 2017; Kulacaoglu and Kose, 2018; Sher et al., 2019). Despite high inpatients prevalence rates, according to several clinicians, BPD is often overlooked and incorrectly diagnosed or underdiagnosed in different mental health treatment centers globally (Leichsenring et al., 2011; Ellison et al., 2018; Gunderson et al., 2018; Linehan, 2018; Wlodarczyk et al., 2018; Chapman et al., 2019). Borderline personality disorder is often mistakenly diagnosed as bipolar disorder, resulting in maltreatment (Fornaro et al., 2016; Marchetti et al., 2021).

Various scales have been developed, following the DSM criteria for BPD, such as the Clinical Global Impression Scale for Borderline Personality Disorder (CGI-BPD), the Zanarini Rating Scale for Borderline Personality Disorder (ZAN-BPD), and the Borderline Personality Disorder Severity Index (Arntz et al., 2003; Zanarini et al., 2003; Perez et al., 2007; Storebø et al., 2018). A basic limitation of these scales is the administration time involved as well as the expertise needed to administer and interpret the scores (Pfohl et al., 2009; Furnham et al., 2014; Esguevillas et al., 2018; Reyes-López et al., 2018). Hence, other instruments were designed for the same purpose, such as the 95- and 23-item Borderline Symptom List (BSL-95; Bohus et al., 2009), the Borderline Personality Features Scale-11 (BPFS-11; Sharp et al., 2014), and the 15-item Borderline Evaluation of Severity Over Time (BEST-15; Pfohl and Blum, 1997; Pfohl et al., 2009). However, the BSL and BEST may have limited applicability in measuring the severity level of BPD (Hafsa et al., 2021; Rashid et al., 2021; Saif et al., 2021; Sarfraz et al., 2021; Toqeer et al., 2021).

Although an Urdu-translated brief measure of borderline personality features exists, its psychometric properties have only been ascertained in adolescents (Bibi and Kazmi, 2021). The drawbacks in previous instruments led to the impetus to create another instrument to assess BPD based on DSM-IV criteria (i.e., McLean Screening Instrument for BPD) (Zanarini et al., 2003).

This scale has been validated for various samples and languages (Wang et al., 2001; Gardner and Qualter, 2009; Leung and Leung, 2009; Melartin et al., 2009; Kröger et al., 2011; Patel et al., 2011; Noblin et al., 2014; André et al., 2015; Soler et al., 2016; Keng et al., 2019). Nevertheless, no study performed the psychometric validation of the Urdu translation of this scale. This suggested the need to validate this scale on Urdu-speaking samples.

Urdu is the national language of Pakistan and is spoken by approximately 60–80 million people (Aqeel and Ahmed, 2017; Choudhry et al., 2018; Barki et al., 2020). It is graded as the fifth frequently spoken language, comprising 4.7% of the entire world population and is spoken typically in Pakistan, South Asia, and India (Mesthrie, 2009; Choudhary, 2017; Barki et al., 2020). With this background, the present study aimed to assess the Urdu-language validation and reliability of the McLean Screening Instrument for Borderline Personality Disorder (MSI-BPD) among individuals with cardiac problems because such problems are common in Pakistan with an average of one in every fourth person suffering from some sort of cardiovascular issues (Jafar et al., 2005). With most of the population not well-versed in the English language, it is difficult to accurately assess BPD in individuals.

The present study sample was limited to individuals with cardiac problems as the main study aimed to assess the prevalence of BPD among individuals with cardiac problems. Several studies have suggested a possible linkage between cardiac problems and BPD (Moran et al., 2007; Lee et al., 2010). Likewise, few studies have also suggested that individuals having BPD are at a higher risk of life-threatening issues due to their associated heart conditions, thus timely screening is important for the well-being of such individuals (Carr et al., 2018; Videler et al., 2019) and to provide the appropriate treatment. For this very reason, it was important to have a translated and validated instrument to assess BPD in individuals with cardiac problems in Pakistan, as the absence of a diagnosis of BPD, despite having its symptoms, could be detrimental to their physical and mental health.

## Methodology

### Research Design

Based on a cross-sectional design, the pilot and main study recruited the participants through the purposive sampling technique. This study was conducted in two phases: (1) pilot, and (2) main study phase. During the pilot investigation, the standard back-translation method was utilized to evaluate the cross-language validation as well as test–retest reliability of the MSI-BPD in the Pakistani population (Anderson and Brislin, 1976; Hambleton, 1994). The main study was conducted to assess the Cronbach alpha reliability and factorial validity of the MSI-BPD through confirmatory factor analysis (CFA) and Pearson correlation analyses.

### Participants

For initial cross-language validation, in the pilot phase, 30 participants, including men (*n* = 15) and women (*n* = 15), with an age range of 16–23 years (*M* = 18, *SD* = ±5.4), were recruited. The participants were enrolled in undergraduate courses at the Department of Psychology, Foundation University Islamabad, Pakistan. These participants were bilingual and knew English as well as the Urdu language. The principal reason for selecting these participants as an initial sample was to evaluate the cross-language reliability and validity of the MSI-BPD.

Afterward, in the main study, a sample of 150 individuals with cardiac problems (men = 75 and women = 75) and an age range of 23-78 years (*M* = 55 years, *SD* = 10.6 years), was selected, using purposive sampling. The sample size was considered suitable as past validation studies have recommended 5–10 persons per scale item (Nunnally, 1978; Cortina, 1993; Lai et al., 2011; Choudhry et al., 2018; Barki et al., 2020; Shuja et al., 2020a).

The participants were recruited from the Cardiac Center, Military Hospital Rawalpindi, and Pakistan Institute of Medical Sciences (PIMS), Islamabad, Pakistan, from January 2016 to August 2017. The sample was selected in line with the main topic of the study, which was to assess the prevalence of BPD in Pakistani Urdu-speaking individuals with cardiac problems. The assumption for the study was based on the notion that a significant relation between cardiac problems and personality disorders exists (Steptoe and Molloy, 2007; Bishop, 2016; Sahoo et al., 2018).

#### Inclusion Criteria

The inclusion criteria for the participants selected for the study were individuals: (1) diagnosed with cardiac problems (such as congenital heart disease, aorta disease, deep vein thrombosis, cardiomyopathy, etc.); (2) inpatients at the time of data collection; (3) receiving treatment in the hospital due to cardiac problems, and (4) agreed to be part of the study.

#### Exclusion Criteria

Similarly, individuals were excluded if (1) their medical history suggested the presence of any comorbid chronic physical condition, such as arthritis, chronic pain, chronic headache, and respiratory problems, etc.; and (2) they refused to take part in the study. A total of nine participants refused to be part of the study (*n* = 4 men, and *n* = 5 women) because of not feeling well, not being interested in the study, etc.

Moreover, a certified clinical psychologist applied the Mini-Mental Status Examination (MMSE) to assess cognitive functioning of those participants, who agreed to be part of the study. This was done to check if the participants were in an optimal cognitive capacity to respond to the items of the MSI-BPD, as they were all inpatients receiving treatment. Nevertheless, no participant was found to have suboptimal or affected cognitive functioning.

### Instruments

#### McLean Screening Instrument for Borderline Personality Disorder

The MSI-BPD (Zanarini et al., 2003; Melartin et al., 2009) is a 10-item instrument used to screen for BPD. The last two items of this instrument assess paranoia and dissociation symptoms in adults (Melartin et al., 2009). Each item of this instrument is rated on a dichotomous scale with 1 corresponding to “present” and 0 corresponding to “absent.” The total score ranges from 0 to 10, and high scores reveal the presence of BPD (Zanarini et al., 2003). The MSI-BPD has illustrated satisfactory reliability and validity (Zanarini et al., 2003). In the current inquiry, the Cronbach alphas were α = 0.78 for BPD, and α = 0.65 for paranoia and dissociation symptoms, respectively. The past empirical evidence has suggested the score of ≥7 as a useful clinical cutoff score in predicting BPD among adults (Zanarini et al., 2003; Chanen et al., 2008; Patel et al., 2011).

#### Translation and Adaptation of MSI-BPD

The standard back-translation method was used, and the translation and adaption were completed in four steps: (1) forward translation, (2) adaption and translation, (3) back translation and committee approach, and (4) cross-language validation. The objective of this study was to acquire a theoretically sound as well as linguistically and culturally appropriate Urdu MSI-BPD. The principal emphasis was on cross-cultural and theoretical correspondence instead of linguistic and word-to-word correspondence. This aim was accomplished using the forward- and back-translation techniques (Anderson and Brislin, 1976).

#### Stage 1: Forward Translation

It was used to ensure the quality of translation and adaptation. A panel of three psychologists, working in public sector hospitals, was selected based on the following criteria: (1) expertise in both languages (i.e., the English and Urdu language), (2) knowledge about both cultures, (3) expertise in the subject matter, and (4) proficiency in verified item writing. The bilingualism of the translators was ensured, and the instrument was handed over to them (Hambleton and Patsula, 1999; Shuja et al., 2020a).

#### Stage 2: Back Translation

In this stage, the instrument was translated back to the original language with the assistance of bilingual translators who had initially translated it into the English language. This method assisted in ensuring the accuracy and efficacy of the instrument translated into Urdu. Both forward- and back-translated versions were found similar regarding clarity and comprehensibility of items (Anderson and Brislin, 1976; Shuja et al., 2020b).

In the initial step of back translation, three bilingual professionals translated the instrument from the Urdu to the English language for equivalence purposes of both original and target versions. The focus of back translation was on theoretical and cultural correspondence contrary to linguistic correspondence. After back translation, inconsistencies were resolved through mutual consensus in a team of subject matter experts (SME). The final items, matching the original instrument as well as cultural correspondence, were retained. The original English version of the MSI-BPD was not shown to the bilingual professionals chosen for back translation. Lastly, the Urdu-translated items were organized as in the original measure.

#### Stage 3: Committee Approach or Reconciliation Session

A commission of SMEs, comprising two psychology lecturers and a graduate-degree-level student, critically inspected the translated versions and resolved any disagreements based on the forward translation and the prevailing or equivalent prior versions. The commission associates examined each translated item and chose the best items for the final scale through mutual consensus. This was done to evaluate the content validity of the translated instrument.

#### Stage 4: Cross-Language Validation

Every language does not have an equivalent share in linguistic resources and instrument advancement; hence, to select an appropriate language according to this context, the English language was chosen. English is a source-rich language and accessible to manuscript examination instruments. For additional evaluation of the translated version in any language, cross-language validation was performed to ensure the efficacy of the Urdu MSI-BPD.

### Procedure

Initially, the present study was approved by the Ethical Committee of the Psychology Department, Foundation University Rawalpindi Campus (FURC), Pakistan with IRB No. 34725ECI-4. Additionally, formal permissions were all taken from the authors of the MSI-BPD as well as concerned authorities of the hospitals before beginning the study.

For the language validation in the pilot phase, 30 participants from FURC University, fluent in both the Urdu and English language, were recruited and further divided into two groups, each group consisting of 15 participants. Both groups were given the Urdu-translated as well as the original English versions of the MSI-BPD to evaluate cross-language validation. This procedure was repeated on the same participants after a gap of 15 days (i.e., after about 2 weeks). This process was used to determine the psychometric properties of both the Urdu and English versions of the MSI-BPD.

Likewise, for the main study, a sample of 150 participants based on the inclusion criteria was recruited from various hospitals in Rawalpindi and Islamabad City. Potential participants who fulfilled the criteria were approached and invited to participate in the current study. The participants were not offered any financial or other kinds of compensation as this was not a funded project. Those who volunteered to participate were given the questionnaire forms with all the necessary information. After reading the explanatory statement, the participants signed the informed consent form. Confidentiality of data was ensured, and anonymity of identities of the participants was maintained. The collected data were then coded and analyzed, using the Statistical Package for Social Sciences (SPSS version 20, IBM Corp., Armonk, NY, USA) and structural equation modeling (SEM).

### Data Analysis Plan

Statistical Package for Social Sciences version 20 was used to analyze indispensable inter-item total correlation, test–retest reliability, and factorial validity of the Urdu MSI-BPD (Dunkley et al., 2006). Confirmatory factor analysis was used to examine the goodness-of-fit (GFI) index of the unique and the implicit structure of the MSI-BPD. Confirmatory factor analysis was performed through SEM. The robust correction method with maximum likelihood was used to amend the distribution issues in the data set. The rules used to determine the fit index of the CFA models to the data set in the present study consisted of root mean square error of approximation (RMSEA) <0.08 and GFI or comparative fit index (CFI) >0.90 as well as a range of CFI and GFI coefficient values between 0 and 1. These fit parameters and the χ^2^ were preferred as earlier research has confirmed their stability and performance (Bentler and Bonett, 1980).

## Results

### Results of Pilot Study

Table 1 shows the Cronbach's alpha coefficient of 0.79. Furthermore, the test re-test reliability method showed an association of *r* = 0.95, indicating that, in the pilot study, the Urdu MSI-BPD had a good overall internal consistency. The test–retest analysis was conducted, following an interval of 2 weeks in a subsample of 30 students with adequate results (*r* = 0.95; *p* < 0.000). Cronbach's alpha reliability of the Urdu MSI-BPD was evaluated by test–retest reliability analysis, and Pearson correlation analysis was performed. Pearson correlation for the total scale was 0.945, indicating a good test–retest correlation.

**Table 1:** Mean and standard deviation, Pearson product-moment correlation coefficient, test–retest reliability of both the Urdu and English versions of the McLean Screening Instrument for Borderline Personality Disorder (MSI-BPD) (*N* = 30).

**MSI-BPD**	**Test**	**Retest**	**Correlation**	***p*-value**
	**(Urdu)**	**(English)**	**coefficients**	
Mean	16.4	16.46	0.95	0.000
SD	2.66	2.56		
Range	0–10	0–10		
Item–total correlation	0.38–0.78	0.25–0.68		
A	0.79	0.77		

*MSI-BPD, McLean Screening Instrument for Borderline Personality Disorder*.

### Results of the Main Study

Table 2 indicates the item-total correlation for the 10 items of the Urdu MSI-BPD. It is evident from the results that most of the items are positively associated with the total scores, demonstrating a high internal consistency of the instrument. The item-total correlations of most of the scale items suggest preliminary reliability of the instrument for the study.

**Table 2:** Item total correlation for the Urdu MSI-BPD (*N* = 150).

**Urdu MSI-BPD**	***r***
1	0.823^**^
2	0.622^**^
3	0.567^**^
4	0.342^**^
5	0.714^**^
6	0.688^**^
7	0.518^**^
8	0.545^**^
9	0.446^*^
10	0.603^**^

* *p ≤ 0.05*,

** *p ≤ 0.01, r = Pearson correlation coefficient*.

**Figure 1:**
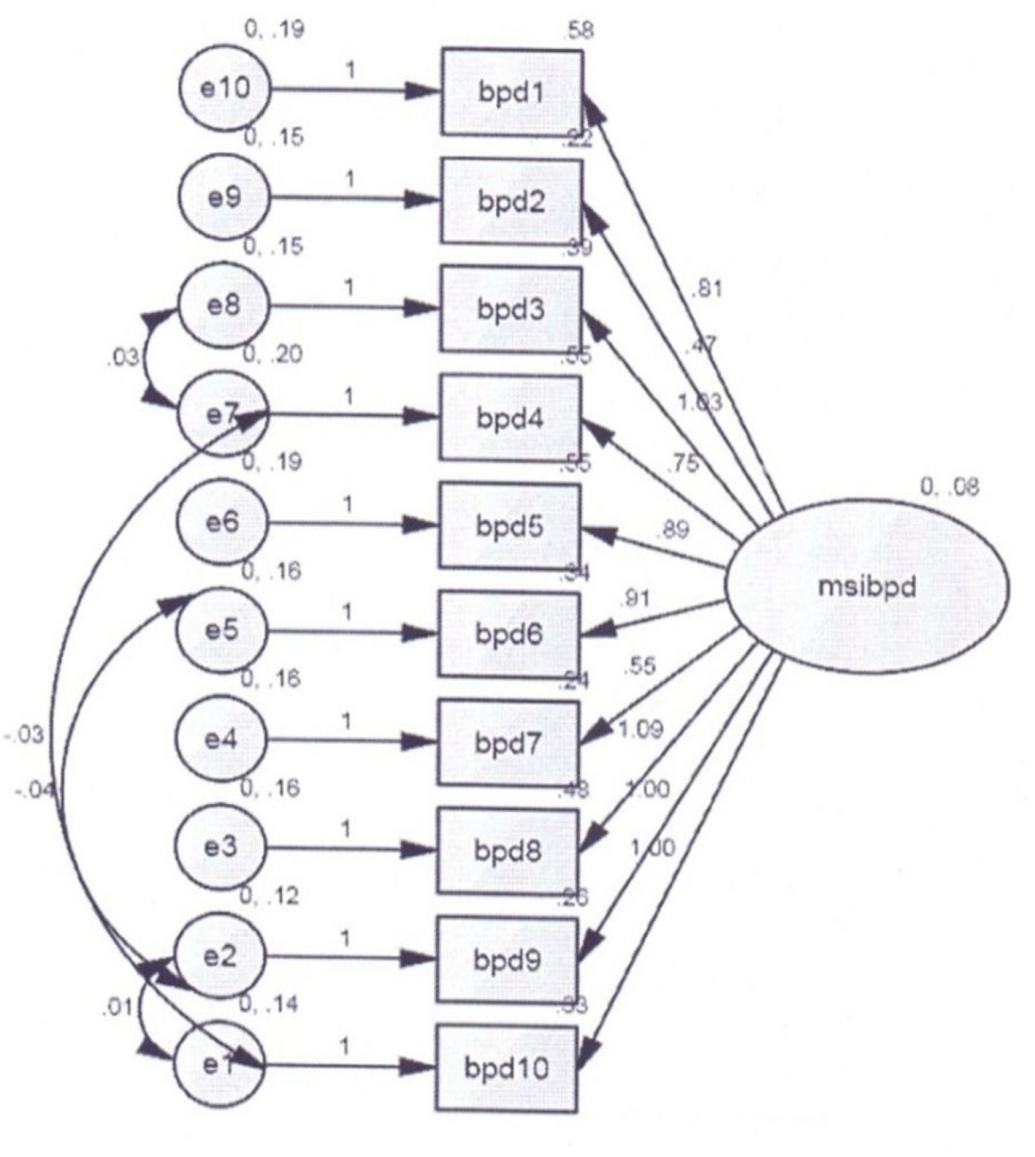
Confirmatory factor analysis for the Urdu McLean Screening Instrument for Borderline Personality Disorder (MSI-BPD).

Table 3 indicates that the indices of the model are perfect and fit for the original factor structures of the MSI-BPD [χ^2^ = 41.85; χ^2^/*df* = 31; *CFI* = 1.35; *IFI* = 0.95; *TLI* = 0.95; *RMSEA* = 0.05, 90% *CI* = (0.14, 0.13); *ECVI* = 0.72, 90% *CI* (0.06, 0.05)] in Pakistan context. It shows the high factorial validity of the Urdu MSI-BPD among individuals with cardiac problems in Pakistan. The modification indices were used to examine and improve the model in the present study. The model produced fit statistics of MSI-BPD in individuals with cardiac problems, and the modification indices recommended that the largest following reduction in the chi-square statistic would be created by permitting the residuals of items seven with eight or one as well as one with two to covary. This suggestion was examined and used to create enhanced GFI in the present model (see Figure 1). Few studies have recommended that the practice of drawing multiple covarying residual variances may be problematic, particularly across factors and items (e.g., Cudeck et al., 2001). Hence, in the present study, the residual term was used because of the possibility of some concepts of overlap of items in a single factor.

**Table 3:** The fit indices of the model of the Urdu MSI-BPD (*N* = 150).

**Scale and factor**	**χ*^**2**^***	***df***	**χ*^**2/**^df***	***CFI***	***IFI***	***TLI***	***RMSEA***	***RMSEA 90% CI***	***ECVI***	***ECVI 90% CI***
Urdu MSI-BPD (10 items)	41.85	31	1.35	0.95	0.95	0.93	0.05	(0.14,0.13)	0.72	(0.6,0.5)

*CFI, confirmatory factor index; IFI, incremented fit index; TLI, Tucker Lewis index; RMSEA, root mean square error of approximation; ECVI, expected cross validation index*.

Table 4 illustrates that there is no item negative estimate in the Urdu MSI-BPD. Factor loading indicate the strength of the associations between the items in terms of shared variance and construct. Standardized regression coefficient scores closer to 1.0 value are considered best and fit. Current study scores ranged from 0.32 to 0.61, which are indicative of the significant GFI of the model.

**Table 4:** Factor loading of confirmatory factor analysis (CFA) for the Urdu MSI-BPD in individuals with cardiac problems (*N* = 150).

**Items no**.	***B***	***S.E*.**	**β**	***p***
10	1.00	–	0.59	<0.001
9	0.99	0.17	0.63	<0.001
8	1.08	0.20	0.61	<0.001
7	0.55	0.15	0.36	<0.001
6	0.92	0.19	0.54	<0.001
5	0.89	0.18	0.50	<0.001
4	0.75	0.19	0.42	<0.001
3	1.03	0.19	0.59	<0.001
2	0.47	0.14	0.32	<0.01
1	0.81	0.18	0.46	<0.001

*SE, standard error*.

## Discussion

Borderline personality disorder is a prevailing and common psychiatric problem often disregarded in treatment settings and affects approximately 0.7–5.9% of the normal populace (Lenzenweger et al., 2007). BPD is underdiagnosed and unclear in both practice and clinical settings (Dhaliwal et al., 2020). One better way toward improving clinical recognition of BPD is the administration of screening tools (Zimmerman, 2017). Therefore, the current study sought to evaluate the psychometric properties of the Urdu-translated version of the MSI-BPD in an Urdu-speaking sample of individuals with cardiac problems in Pakistan. The findings of the present study illustrate that the one-factor model fits the data. These findings of the one-factor structure of the Urdu MSI-BPD are reliable in a sample of people living with cardiac problems (Zanarini et al., 2003; Leung and Leung, 2009; Soler et al., 2016; Keng et al., 2019).

In harmony with a past study, the Urdu MSI-BPD revealed better internal consistency (Keng et al., 2019). The whole MSI-BPD and its items showed significant positive associations in the present study. Also, test–retest reliability coefficient, after 2 weeks' interval, with a sample of 30 university students, provided significant association between the Urdu and the English versions of the MSI-BPD. Moreover, the findings of the CFA supported the application of a one-factor structure in a sample of people with cardiac problems in Pakistan.

The study attempted to evaluate the validity and reliability of the Urdu-translated version of the MSI-BPD among individuals with cardiac problems. This scale can be used as a preliminary screening tool to measure BPD in hospital settings, which is commonly ignored in Pakistan due to the non-availability of a valid instrument. Furthermore, the current study attempted to evaluate the reliability and factorial validity among individuals with cardiac problems as this population is more vulnerable to life-threatening issues (Carr et al., 2018; Videler et al., 2019). Previous literature also suggests a significant relationship between cardiac problems and personality disorders (Steptoe and Molloy, 2007; Bishop, 2016; Sahoo et al., 2018). For this reason, and to assess the prevalence of BPD in cardiac patients, the present study was performed.

The findings of the current study are in harmony with the results of prior studies conducted on various samples, such as the Spanish (Soler et al., 2016), Dutch (André et al., 2015), Chinese (Leung and Leung, 2009), Malay (Mazlan and Ahmad, 2012), Hindu (Choudhary, 2017), and Russian (Tucker et al., 2016) studies. For the final evaluation of current findings, it could be considered that CFA is the best way to establish factorial validity in any population (Hu and Bentler, 1999).

The Urdu and English versions of the instrument had an appropriate level of reliability (i.e., α = 0.739, α = 0.770, respectively). These findings suggest that both versions of the questionnaire are reliable for screening BPD. The present study extends the body of literature on instruments used to screen BPD and suggests the use of the MSI-BPD as a screening instrument for adults with cardiac problems, resembling the original study (Zanarini et al., 2003). The original instrument was also initially formulated for use with the adult population (Zanarini et al., 2003).

The study has demonstrated a relationship between the English and Urdu versions of the MSI-BPD. The significant values of test–retest correlation suggest that both versions correlated with one another. The present study has shown a high internal consistency of the Urdu version of the instrument through inter-item correlation. Furthermore, all the items of the Urdu version exhibited significant positive correlations. This study has evidenced the utility of the MSI-BPD among adult cardiac inpatients. The findings can help in preventing the misdiagnosis of bipolar disorder among individuals with BPD as BPD is often mistakenly diagnosed as a bipolar disorder causing maltreatment (Fornaro et al., 2016; Marchetti et al., 2021).

### Strengths and Limitations

This study is vital as it indicates the possible use of the MSI-BPD as a screening tool for adults with cardiac problems. It is important to appraise its use with various populations as a comparatively recent screening tool. The findings of the present study are an attempt to add to the directory of validated populations for which the MSI-BPD can be used. Additionally, the present study, by having a gender-balanced sample, also suggests that the tool can be applied to both male and female adults with cardiac problems. Nevertheless, as a preliminary study, the evaluated translated version of the scale has shown appropriate reliability and factorial validity.

However, there are also some limitations. Most importantly, although the scale has displayed good reliability and factorial validity, future studies should evaluate other validities such as convergent, divergent, and predictive validities. Currently, the translated instrument only displays limited evidence of being able to screen people suffering from BPD with preliminary factorial validity. However, more research needs to be done, as, in its current form, the scale does not have any convergent validity (i.e., all the interrelated items measure the desired construct-BPD). For this purpose, another scale assessing any features of BPD should be administered to confirm the construct validity. Likewise, another scale that can establish its divergent validity should also be applied simultaneously along with the translated MSI-BPD. This should be done to ensure that the scale measures only BPD and not any other construct (e.g., psychopathology). Hence, future studies may use translated measures of constructs that assess any features of BPD (Bibi and Kazmi, 2021).

Moreover, the predictive validity of the current translated scale should also be established to ascertain if the items can predict the prevalence of BPD over time. Additionally, since the scale is of diagnostic nature, it would have been appropriate to develop its sensitivity and specificity. However, due to the limitations of the present study as being more of a pilot study for constructing a translated version of the MSI-BPD, no additional samples were recruited. Nevertheless, this study should be replicated by recruiting more samples to develop appropriate cut-off scores along with the sensitivity and specificity of the scale. This would be an additional strength to the translated scale and further validate the scale as a diagnostic instrument.

Furthermore, a sample of individuals with and without a clinical diagnosis of BPD can be recruited to evaluate the specificity and sensitivity of the translated instrument. As the MSI-BPD is a self-reporting questionnaire, various biases may exist, such as social desirability or social conformity. Such biases can be reduced in future studies by employing a combination of prevention and detection methods. Additionally, in this study, the test–retest reliability was assessed based on a short span of time and a considerably small sample size. Hence, future studies should evaluate test–retest reliability after longer spans of time with larger samples.

Also, the information related to various sociodemographic factors, such as marital status, work status, whether the participants had undergone surgery, or were simply diagnosed with cardiac problems, the duration of stay in the respective hospitals, etc., was not obtained at the time of collecting data. This was due to limitations including restrictions on part of the hospitals from where the data were collected and reluctance of the participants in providing such details. These factors may have influenced the findings of the study; hence, future studies should collect information related to sociodemographic characteristics.

## Conclusion

This study suggests that the Urdu version of the McLean Borderline Personality Disorder Scale (MSI-BPD) can be used as an effective instrument to screen BPD in Pakistani adult individuals with cardiac problems. The easy scoring and implementation of the scale make it a useful tool for primary screening at hospitals and mental health clinics. However, the study also recommends further validation of the scale, along with ascertaining its sensitivity and specificity. Moreover, the translated scale should be further applied to other samples in the future to evaluate appropriate norms for various populations. In its current form, the scale serves as a basis for the Urdu version of the MSI-BPD with appropriate reliability and factorial validity. The study also sheds light on the existence of a high number of BPD in individuals with cardiac problems that should be further explored in subsequent studies.

## Data Availability Statement

The datasets generated for this study are available on request from the corresponding author.

## Ethics Statement

This study involving human participants, was reviewed and approved by the Ethical Committee of the Psychology Department, Foundation University Rawalpindi Campus (FURC), Pakistan with IRB number 34725ECI-4. Additionally, formal permission was taken from the authors of the MSI-BPD before beginning the study. The patients/participants provided their written informed consent to participate in this study.

## Author Contributions

KM, FRC, and MA were involved in conceptualization, data curation, formal analysis, and original draft writing. TR, KS, and FB were involved in finalizing the methodology, data collection, formal analysis, and draft writing. All the authors were involved in approving the final version of the manuscript.

## Conflict of Interest

The authors declare that the research was conducted in the absence of any commercial or financial relationships that could be construed as a potential conflict of interest.

## Publisher's Note

All claims expressed in this article are solely those of the authors and do not necessarily represent those of their affiliated organizations, or those of the publisher, the editors and the reviewers. Any product that may be evaluated in this article, or claim that may be made by its manufacturer, is not guaranteed or endorsed by the publisher.
